# Genome–phenome association prediction using weighted deep matrix factorization with a multisource graph attention network

**DOI:** 10.1002/qub2.70044

**Published:** 2026-06-18

**Authors:** Ran Duan, Wei Cao, Maozu Guo, Le Tian, Lingling Zhao

**Affiliations:** ^1^ School of Intelligence Science and Technology Beijing University of Civil Engineering and Architecture Beijing China; ^2^ Beijing Key Laboratory of Super Intelligent Technology for Urban Architecture Beijing University of Civil Engineering and Architecture Beijing China; ^3^ School of Computer Science and Technology Harbin Institute of Technology Harbin China

**Keywords:** deep matrix factorization, genome‐phenome association, graph attention network, multi‐omics data

## Abstract

Genome–phenome association (GPA) prediction can broaden the understanding of biological mechanisms underlying complex phenotypic traits (e.g., diseases and agronomic traits). Traditional deep matrix factorization (DMF)‐based GPA methods can integrate multiple data types and uncover nonlinear associations but often rely on low‐dimensional matrix representations, making it challenging to capture intricate node interactions under sparse data conditions. Existing approaches also inadequately integrate multi‐omics data and frequently neglect local neighborhood (LN) and graph structural information, particularly when handling incomplete or low‐quality attribute data. Furthermore, most methods fail to effectively address the scarcity of plant phenotype data. We propose GDMF, an end‐to‐end GPA prediction framework that integrates a multisource graph attention network (GAT) with weighted DMF. By constructing a heterogeneous molecular network, GDMF unifies multi‐omics data and utilizes GAT’s attention mechanism to process multisource associations (both cross‐type and intra‐type node interactions), thereby enhancing the capture of LN structures while preserving critical node information. The weighted DMF component dynamically assigns importance weights to global relationship matrices, enabling robust modeling of complex gene–phenotype interactions even with sparse and noisy attribute data. To mitigate the scarcity of plant phenotype data, GDMF incorporates textual semantic similarity of trait ontology terms to enrich phenotypic attribute representations. Extensive experiments on maize and human datasets demonstrate that GDMF significantly outperforms competitive methods, validating its efficacy and superiority in GPA prediction.

## 1 INTRODUCTION

The genome of an organism is its complete set of genetic material, whereas the phenome refers to the observable traits under specific conditions [1, 2, 3]. Genome–phenome association (GPA) analysis predicts phenotypic traits based on genomic profiles and environmental influences. High‐throughput technologies and multi‐omics (integrating genome, proteome, transcriptome, epigenome, metabolome, and microbiome) enable comprehensive analysis of biological systems [4]. GPA links multiple omics layers to reveal genetic associations between genes and phenotypes, aiding in disease research, breeding, and trait development. Traditional GPA methods, such as quantitative trait locus mapping and functional studies, are limited by long cycles, high costs, and low precision.

With the development of systems biology and the accumulation of multisource information, researchers have found that genes with similar structures and functions often participate in the same biological pathways [5, 6]. Early methods relying on single‐omics data could only provide a one‐sided view of biological processes, leading to incomplete understanding of biological mechanisms [4, 7]. Many researchers have proposed methods based on heterogeneous networks to better elucidate the genetic mechanisms underlying organisms [8, 9]. However, biological processes are highly complex, and prediction methods based on single‐ or two‐omics data fail to consider the impact of other molecular layers, hindering a comprehensive understanding of biological systems [10]. Multi‐omics‐based GPA methods provide a more comprehensive understanding by integrating diverse datasets, although challenges such as data heterogeneity and missing values remain. For example, Feng et al. [11] integrated phenomics, metabolomics, and genomics data to decode the genetic basis of rice metabolism, whereas Mowgli used non‐negative matrix factorization (NMF) to integrate single‐cell multi‐omics data, enhancing cell clustering and biological interpretability [12].

With the increasing understanding of gene function and biological networks, and the growing recognition of the powerful capabilities of machine learning and deep learning in identifying complex nonlinear patterns [4], GPA analysis has come to be viewed as a link prediction problem. To solve this problem, researchers have proposed various computational methods, mainly falling into three categories: network propagation, matrix factorization (MF), and graph neural network (GNN) methods. These methods are not only capable of handling large scale multi‐omics data but also possess strong generalization abilities, enabling accurate predictions on unseen data.

In network propagation methods, random walk algorithms and label propagation algorithms are two typical examples. For instance, the BiRW method [13] explores the potential paths between genes and phenotypes through independent random walks on gene interaction networks and phenotype similarity networks, incorporating these paths’ correlation for prediction. For instance, Chen et al. [14] incorporated data from lncRNA, miRNA, and disease levels to construct a heterogeneous network and used label propagation strategies to predict the association between miRNA and disease.

MF methods map genes, phenotypes, and other molecules to latent low‐dimensional feature spaces and then predict GPA by reconstructing molecular correlation matrices based on low‐dimensional representations. For instance, Wang et al. [15] incorporated multiple association matrices into a low rank representation matrix through collaborative factorization and optimized weights to predict the association between lncRNA and diseases. Further, Yu et al. [16] used matrix tri‐factorization to incorporate attribute data and multiple molecular networks. Although these methods efficaciously incorporate different data types and learn low‐dimensional representations of molecules, they are insufficient in exploring nonlinear associations in heterogeneous networks. Deep matrix factorization (DMF) [17], a subfield of deep learning, has been widely applied in biological network link prediction owing to its ability to uncover deep semantics and nonlinear complex associations in data. For instance, Chen et al. [18] proposed a deep non‐negative MF method to learn non‐negative feature representations of nodes for efficacious link prediction in graphs. Similarly, Lu et al. [19] combined explicit and implicit feedback, using DMF and multi‐layer neural networks to predict the potential associations between circRNA and diseases.

To enhance the robustness, interpretability, and topological preservation capability of MF methods in biological networks, a series of regularization‐enhanced variants have been proposed. Graph‐regularized NMF preserves the local neighborhood (LN) structure of samples in low‐dimensional representation learning by introducing graph Laplacian regularization [20]. Building on this, adaptive mechanisms (e.g., dynamic adjustment of graph structure) and self‐paced learning strategies have been incorporated to alleviate dependence on predefined graphs and improve stability in noisy data [21]. To enhance sparsity and noise resistance, graph NMF with alternative smoothed *L*_0_ regularizations imposes structured sparsity constraints on the factor matrices to highlight key associations [22]. Furthermore, for multisource data integration, multi‐view graph regularized NMF learns consistent representations across views through a deep architecture with consensus constraints [23], while robust matrix completion via capped nuclear‐norm regularization improves robustness to outliers through a more accurate rank approximation [24]. However, most of these methods still rely on manually constructed static graphs and a linear decomposition framework, treating graph information merely as a regularization penalty. Consequently, they struggle to adaptively learn non‐linear high‐order interactions in complex heterogeneous networks, which limits their expressive power for multi‐omics GPA prediction.

Graph data can represent associations between entities, while heterogeneous graphs represent the associations between various types of entities. In recent years, GNN, a representative method of graph representation learning, has been rapidly developed and widely applied in graph data including social networks, knowledge graphs, biological networks, and molecular structures. Traditional matrix‐based methods struggle to capture the complex relationships between molecular entities. In contrast, graph‐based models offer a powerful framework that represents molecular entities and their interactions through nodes and edges [4]. For instance, MOGONET [25] utilizes graph convolutional networks (GCNs) to integrate multi‐omics data, enabling patient classification (CLS) and biomarker identification. By constructing modality specific feature graphs for information propagation, GNNs can capture complex associations between different modalities [26, 27], thereby enhancing integration effects. Currently, GNNs are extensively used in GPA prediction. Variants of GNNs, termed GCNs, more effectively utilize omics features and the correlations between samples or data types through the use of similarity networks. These similarity networks impose biologically meaningful structures onto the model, thus offering more interpretable advantages. Furthermore, graph attention networks (GATs) can reveal important patterns and associations hidden in the data by focusing on key nodes and edges in the graph, thereby providing more accurate results in tasks such as GPA prediction [4]. For instance, Ai et al. [28] used multiple GCNs to process data from different sources, learning feature embeddings and reformulating miRNA‐disease association scoring matrices. Moreover, Jose L et al. [29] employed heterogeneous information networks and graph‐based convolutional neural networks to assign scores to phenotype–gene associations and predict potential associations. The GraphCDA method [30] combined GCN and GATs to simultaneously learn feature representations of circRNA and diseases and make association predictions.

The aforementioned methods mainly focus on the association of human transcriptome level molecules with phenotypes. However, for plants, particularly major crops including rice, soybean, and maize, the known GPAs are limited, and computational genetic analysis research on crop GPA is relatively scarce. With the rapid development of expression profiling and metabolomics technologies, multi‐omics data for plants including *Arabidopsis*, maize, and rice are increasingly available [31, 32], driving further advancements in crop genetic intelligence analysis and breeding research based on multi‐omics data. For instance, Tan et al. [33] proposed the iMRW method, which constructs a multi‐omics heterogeneous network by incorporating gene, miRNA, and phenotype nodes. They designed a personalized multi‐element random walk strategy that assigns different walk lengths to nodes of varying importance, thereby predicting the association information between network nodes. Furthermore, Tan et al. [34] introduced the DeepGPA method, which first incorporates multi‐omics associations and attribute data and then utilizes DMF to explore the complex nonlinear features for GPA prediction. Tan et al. [35] presented the WDGPA method, which builds on DeepGPA by incorporating an adaptive strategy to weight the fusion of association and attribute data, and they performed DMF on the heterogeneous network matrix in a coordinated manner to find nonlinear representations of nodes for GPA prediction.

Despite significant efforts to explore potential GPA, several challenges remain, including the underutilization of multisource information and lack of attribute data. First, many computational methods fail to fully utilize trait ontology (TO) terms, as simply considering the parent–child association structure of TO terms is insufficient to capture all information, leading to sparse phenotype data and reducing prediction accuracy. Second, the integration methods of data concatenation and dimensionality reduction may neglect important neighborhood structural information of certain nodes owing to the overall data quality. Furthermore, these methods often depend on multistage processing and some predefined data or manually crafted intermediate results, lacking a complete end‐to‐end method. To address these shortcomings, we propose a method that combines a multisource GAT with weighted DMF, termed GDMF. This method directly models heterogeneous graphs containing multiple types of nodes and edges and effectively integrates multi‐omics data. Using GAT, GDMF captures LN and graph structural (GS) information from nodes, retaining important node information to generate initial feature representations. Finally, GDMF incorporates global information through weighted DMF and predicts GPA in an end‐to‐end method. In addition, this method also applies to processing the descriptive texts of other ontology terms or other textual information. To evaluate the efficacy of GDMF, we compared it with six state‐of‐the‐art methods, using five‐fold cross‐validation. The experimental results show that GDMF outperformed existing methods in prediction accuracy and generalization ability.

## 2 RESULTS

This section provides a detailed demonstration of the outstanding performance of GDMF on the human and maize benchmark datasets. First is a brief overview of the GDMF method, followed by a detailed description of the data collection and preprocessing process, benchmark methods, and evaluation metrics. The experimental results show that GDMF has achieved significant improvements in multiple key metrics, comprehensively surpassing the existing six advanced methods, which fully proves its powerful ability to integrate multi‐omics and handle complex biological data. In particular, in the de novo prediction scenario of the human and maize datasets, GDMF still shows the best performance, indicating that even under data‐scarce conditions, GDMF can still accurately predict the associations between unknown genes and phenotypes. Through specific case analysis, GDMF has successfully predicted the genes associated with key maize phenotypes, providing a powerful tool for plant breeding.

### 2.1 Overview of GDMF

GDMF is an innovative application of GNN and DMF in the field of plant data and is also applicable to human data. As a method for predicting GPAs based on the integration of multi‐omics data, the GDMF framework includes four core modules: Construction of a multi‐omics heterogeneous information network, enhancement of TO text data, fusion of multisource information, and weighted prediction, as shown in Figure 1.

**FIGURE 1:**
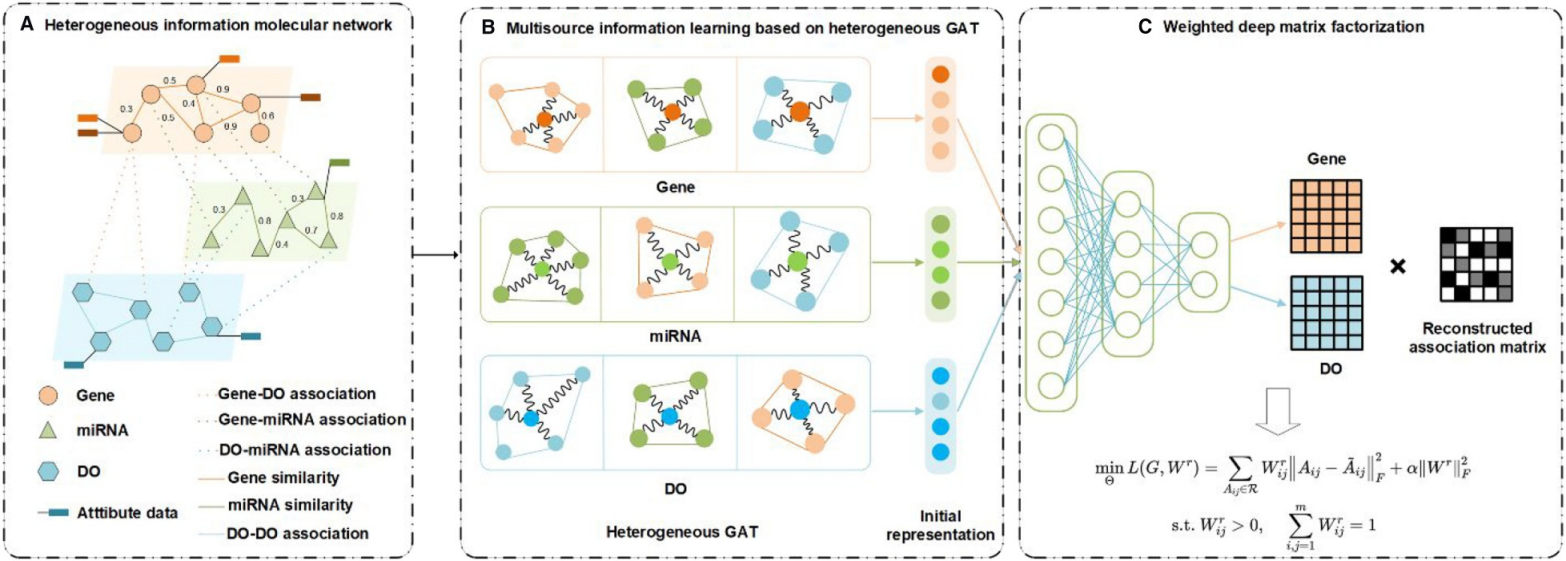
Overview of GDMF. (A) Construct a heterogeneous molecular network consisting of three types of nodes: Gene, miRNA, and TO terms, with nodes connected through different types of edges. (B) Utilize a hierarchical GAT to process multisource information in the network, learning attention weights between nodes to capture LN information. Independent GAT layers are applied to process each node type, preserving the information of key nodes and generating an initial feature representation for each type of molecule. (C) Optimize the low‐dimensional representations of each molecule type through DMF, and incorporate global information through an adaptive strategy, enhancing model performance and generating the corresponding association matrix. DMF, deep matrix factorization; DO, disease ontology; GAT, graph attention network; GDMF, an end‐to‐end GPA prediction framework that integrates a multisource GAT with weighted DMF; LN, local neighborhood; TO, trait ontology.

In constructing the heterogeneous information molecular network, GDMF integrates three types of nodes—genes, miRNAs, and TO terms—and standardizes the representation of associations and attribute information using different types of relationships and attribute data, as illustrated in Figure 1A. Subsequently, by employing the sentence‐BERT (SBERT) [36] technique to process the textual descriptions of TO terms and calculate the text semantic similarity between TO terms, GDMF enriches the attribute data of TO and accurately delineates the associations between TO terms. Based on the hierarchical graph attention network (H‐GAT), as shown in Figure 1B, GDMF integrates interactions between nodes of the same type and different types through type‐specific attention mechanisms, prioritizes high‐confidence biological interactions, and mitigates the impact of noisy edges using dynamic attention weights. Finally, as depicted in Figure 1C, by leveraging weighted DMF technology to integrate global information from different relational data and employing an adaptive strategy to capture more complex nonlinear structures and global dependencies, GDMF effectively compensates for the shortcomings of GAT in global information fusion. For more details about this method, please refer to Section 4.

### 2.2 Experimental setup

To verify the effectiveness and superiority of the proposed GDMF method in the task of GPA prediction, we designed and conducted a series of rigorous experiments. The following sections will provide a detailed introduction to the experimental settings, results, and analysis processes.

#### 2.2.1 Datasets and preprocessing

To evaluate the performance of GDMF, we considered three types of nodes: genes, miRNAs, and TO terms. We then validated the performance on maize and human datasets. We collected and processed the association matrices for these node types from public databases, with the relational matrices listed in Table 1 and the attribute matrices listed in Table 2.

**TABLE 1:** Details of collected relationship data sources.

Species	Relation	Datasets	Size	Notation	Source
Maize	Association	Gene‐pathway	12,098 × 368	*A*_13_	Tas.hzau.edu.cn
		Gene‐TO	12,098 × 155	*A*_14_	Tas.hzau.edu.cn
		miRNA‐TO	447 × 155	*A*_24_	Generated by $A_{12}^{T}\cdot A_{14}$
		Gene‐miRNA	12,098 × 447	*A*_12_	Pmirex.com/resource
	Interaction	Gene‐gene	12,098 × 12,098	*R*_11_	Maizegdb.org
		miRNA‐miRNA	447 × 447	*R*_22_	Pmirex.com/resource
		TO–TO	155 × 155	*R*_44_	Obofoundry.org
Human	Association	Gene‐pathway	12,396 × 212	*A*_13_	Mirtbase.cukk.edu.cn
		Gene‐disease	12,396 × 290	*A*_14_	Pathwaycommons.org
		miRNA‐disease	550 × 290	*A*_24_	Disgenet.org
		Gene‐miRNA	12,396 × 550	*A*_12_	Cuilab.cn/hmddd
	Interaction	miRNA‐miRNA	550 × 550	*R*_22_	Cuilab.cn/hmddd and constructed by [37]
		Disease‐disease	290 × 290	*R*_44_	Disease‐ontology.org and constructed by [37]

Abbreviation: TO, trait ontology.

**TABLE 2:** Details of collected attribute data sources.

Type	Size	Notation	Feature type	Source
Gene	12,098 × 256	*X*_11_	Gene sequence	Maizegdb.org/
	12,098 × 256	*X*_12_	Gene function profiles	Maizegdb.org/
	12,098 × 225	*X*_13_	Gene expression profiles	Maizegdb.org/
miRNA	447 × 111	*X*_21_	miRNA expression profiles	Pmirex.com/resource
	155 × 128	*X*_41_	TO hierarchical structure embedding	Obofoundry.org and constructed by [38]
TO	155 × 155	*X*_42_	Textual semantic similarity	Obofoundry.org
	155 × 155	*X*_43_	Semantic similarity	Obofoundry.org
Gene	12,396 × 13	*X*_11_	Gene expression profiles (13 samples)	Ebi.ac.uk/gxa/experiments/EMTAB‐4344/
	12,396 × 44	*X*_12_	Gene expression profiles (44 samples)	Ebi.ac.uk/gxa/experiments/EMTAB‐5214/
	12,396 × 53	*X*_13_	Gene expression profiles (53 samples)	Ebi.ac.uk/gxa/experiments/EPROT‐3/
	12,396 × 256	*X*_14_	Gene function profiles	Genontology.org
miRNA	550 × 40	*X*_21_	miRNA expression profiles	Doi.org/10.1186/1471‐21648‐1666
Disease	290 × 256	*X*_41_	DO hierarchical structure embedding	Diseaseontology.org

*Note*: The first 7 rows are attribute data for maize, and the last 6 rows are attribute data for humans.

Abbreviations: DO, disease ontology; TO, trait ontology.

Notably, we obtained the known associations between genes and phenotypes from the study by Pan et al. [39]. This study extracted data from 79 maize and rice association mapping studies, such as genome‐wide association studies, systematically organized the associations between genes and traits, and constructed a large‐scale TO database covering maize (6345 genes) and rice (11,697 genes), with over 100,000 gene‐TO associations. Additionally, by comparing the enrichment analysis of TO and gene ontology (GO), the study found that TO is more phenotypically specific in functional annotation. For example, when analyzing maize height‐related genes, TO can directly associate with phenotypic terms such as “plant height” and “plant morphology”, whereas GO only provides more abstract functional descriptions like “apical cell growth”. This further validates the unique value and significance of TO analysis in phenotypic association studies.

To ensure the independence and integrity of the input data, we obtained datasets from multiple databases and performed independent preprocessing steps for each dataset, including imputation of missing values, normalization, and dimensionality reduction. By doing so, we minimized the potential overlap between different datasets, ensuring the fairness of model training and the reliability of the results. Specifically, we retrieved the known GPAs from the database [39], where matrix $A_{14}$ stores the associations between 12,098 genes and 155 terms. If gene i is associated with term j, then $A_{14}\left(i,j\right)=1$; otherwise, it is 0. We also obtained miRNAs and their target genes from the MaizeGDB database [40], with matrix $A_{12}$ storing the associations between 12,098 genes and 477 miRNAs. If gene i is associated with miRNA j, then $A_{12}\left(i,j\right)=1$; otherwise, it is 0. Given the miRNA target genes, we can derive the miRNA‐TO association network $A_{24}$ by integrating the GPA network $A_{14}$ and the miRNA–target gene network $A_{12}$, that is, $A_{24}=A_{12}^{T}\cdot A_{14}$.

We retrieved 12,098 gene sequences from the MaizeGDB database (version AGPv3.21), computed gene sequence similarities using the basic local alignment search tool, and constructed a gene interaction network based on these similarities. Additionally, we obtained all miRNA sequence information from the PmiREN database (collected on June 17, 2019) [41]. Inspired by the work of Liang et al. [38], we used miRNA sequence data to construct the miRNA interaction matrix for the maize dataset and build the miRNA interaction network. Based on the hierarchical structure of TO, we constructed a TO interaction network representing the parent–child hierarchical relationships between TO terms.

For attribute data, gene sequence features were represented by 4‐mer [42] aggregated tetramer sequences. Each group of four nucleotides in the gene sequence is considered as a combination and mapped to a unique index value using a quaternary numerical system. This process results in a 256‐dimensional frequency vector, where each dimension corresponds to the occurrence count of a specific 4‐nucleotide combination. To mitigate the impact of varying gene sequence lengths on the frequency, the frequency vector is normalized. Gene expression data were obtained from the MaizeGDB database (collected on July 8, 2024), and miRNA expression data were obtained from the PmiREN database (collected on June 17, 2019). The expression data were processed by imputing missing values with the mean and normalizing the data to eliminate technical variations among samples. The gene function map based on GO annotations was subjected to dimensionality reduction using principal component analysis (PCA), with the target dimension set to 256.

TO attribute data include TO hierarchy embeddings, the TO hierarchical structure, and semantic similarities derived from gene annotations and TO text semantic similarity. Specifically, the hierarchical structure matrix of TO was loaded from its ontology file to construct a directed acyclic graph (DAG) of TO. Then, DeepWalk [43] was employed on the DAG to obtain the embedding vectors for each TO term, with the dimension of the embedding vectors set to 128. Based on the work of Yu et al. [44], TO semantic similarity was computed using gene TO annotations and TO hierarchical structures. Given the sparsity of the TO hierarchy matrix due to the limited number of TO terms associated with genes, we enriched the relationships among TO terms by deriving TO text semantic similarity from SBERT‐based calculations of textual similarity between TO terms.

Furthermore, although there may be some overlap between the genes and other input datasets, the TO data are only associated with genes. The GDMF method effectively reduces dependence on a single dataset by integrating multisource data and employing a dynamic weighting strategy. This approach not only enhances the robustness of the model but also ensures the reliability of the results. In human attribute data sources, we integrated three gene expression datasets obtained from independent experimental studies across diverse tissues. Although these datasets vary in their specific gene measurement scopes, they exhibit complementary characteristics that synergistically enable a multidimensional representation of gene expression profiles throughout the human body.

#### 2.2.2 Baselines

We compared our method with six competitive algorithms: BiRW [13], iMRW [33], GraphCDA [30], DMF [17], DeepGPA [34], and WDGPA [35]. BiRW performs random walks on a heterogeneous network composed of GPA matrices, gene similarity matrices, and phenotype similarity matrices to predict GPA. iMRW integrates multi‐relational networks, such as gene–miRNA, gene–phenotype, and miRNA–phenotype networks, and employs personalized random walks to predict GPA. GraphCDA predicts GPA by combining GCN and GAT on a heterogeneous network consisting of GPA matrices, gene expression data, and phenotype feature matrices based on TO term hierarchy embeddings. DMF predicts GPA by mining multilayer nonlinear mappings to reconstruct GPA matrices. DeepGPA and WDGPA incorporate multiple relational and attribute data sources, such as gene expression data, miRNA expression data, and TO embeddings, to predict GPA. In addition to the aforementioned networks, iMRW, DeepGPA, and WDGPA also utilized annotations from GO. The difference between DeepGPA and WDGPA is that the latter performs weighted fusion of multisource data, selectively combining network topology and node attribute information. The key difference between GDMF and DMF, DeepGPA, and WDGPA is that GDMF processes multisource information step by step through an independent GAT to more accurately capture LN and GS information, with less demand for the quality of attribute data. Compared with GraphCDA, GDMF incorporates multi type molecules and their attribute data. The parameters for the comparison methods were fixed to default values in the public shared code or optimized within the ranges specified in the corresponding literature. For GDMF, we first constructed a heterogeneous graph containing various types of nodes and edges and applied the GAT attention mechanism to assign specific weights to each edge type. The exponential linear unit activation function was employed to capture the features of neighboring nodes and the graph structure information. After finding the nodes’ graph representation, we further processed the features through a two‐layer neural network using LeakyReLU as the activation function, ultimately completing the prediction task. For different layers of GAT, the output dimensions could be set according to the specific attribute data’s dimensionality and the relationship data’s richness, with the number of attention heads being set to 6. The hidden layer sizes for DMF were set to $d_{1}=96$ and $d_{2}=40$. The parameter sensitivity analysis will be studied later.

#### 2.2.3 Evaluation metrics

We used the three‐representative metrics (area under the receiver operating characteristic curve [AUROC], area under the precision‐recall curve [AUPRC], and $F_{\max}$) to comprehensively assess the comparative methods. AUROC and AUPRC are the AUROC and the AUPRC at different thresholds, respectively. These metrics are commonly used for binary CLS, with AUPRC being more sensitive to class imbalance. $F_{\max}$ represents the maximum harmonic mean of precision and recall at different thresholds θ∈[0,1] for the predicted GPA matrix:

$$F_{\max}=\max_{\theta \in \left[0,1\right]}\frac{2\times P\left(\theta \right)\times R\left(\theta \right)}{P\left(\theta \right)+R\left(\theta \right)},$$

where P(θ) and R(θ) are the precision and recall at threshold θ, respectively.

### 2.3 GDMF achieves superior performance in GPA prediction

To evaluate the performance of GDMF in identifying potential genes linked to important phenotypes, we carried out five‐fold cross‐validation with 10 repetitions. The GPAs were randomly divided into five equal parts. For each fold, one part served as the test set, whereas the remaining four parts constituted the training set. To ensure the independence of the training and test data and prevent data leakage, we generated an index matrix that included all possible row and column indices of the GPA matrix. These indices were randomly shuffled to guarantee a random split of the data. In each fold, the indices corresponding to the test set were masked (i.e., set to 0) in the training matrix. This ensured that the model was trained exclusively on the training data without any contamination from the test data. The comparative results, shown in Table 3, validate the GDMF design’s rationality. As indicated in Table 3, GDMF achieved the best performance in AUROC, AUPRC, and $F_{\max}$, with iMRW ranking second. This is because, after incorporating multi‐omics data, iMRW captures the neighborhood information of nodes in the heterogeneous graph efficaciously through personalized random walks and, to some extent, avoids excessive propagation of less important nodes. Still, it tends to overlook global information. Contrastingly, GDMF outperformed iMRW by not only capturing LN structures through GAT but also incorporating global information via weighted DMF, highlighting the advantages of combining GAT with DMF. GDMF also outperformed WDGPA and DeepGPA, which further shows the efficaciousness of GAT in supplementing local structural information. Although DMF efficaciously incorporates multi‐attribute and weakly connected data sources, depending only on DMF tends to overlook important nodes owing to noise in global associations. GDMF overcomes this issue. The performance of GDMF was superior to that of DMF, GraphCDA, and BiRW, indicating that the integration of multi‐omics data can efficaciously enhance performance. Additionally, we found that, except for GDMF, other methods, even with good AUROC scores, performed poorly in AUPRC and $F_{\max}$, which mainly stems from the significant class imbalance in the dataset. GDMF applies an attention mechanism to each edge type, ensuring that the model recognizes important nodes that may have poor overall performance, including when certain association or attribute matrices have weak global performance, causing WDGPA to assign them lower weights. This helps avoid ignoring valuable information owing to noise in some attribute matrices, significantly reducing the reliance on the quality of attribute matrices and showing the robustness of GDMF on imbalanced datasets.

**TABLE 3:** Evaluative metrics (mean ± standard deviation) of the comparative methodologies applied to the maize and human datasets.

Methods	Maize			Human		
	AUROC	AUPRC	$F_{\max}$	AUROC	AUPRC	$F_{\max}$
BiRW	0.8106 ± 0.0003	0.5486 ± 0.0025	0.5447 ± 0.0010	0.8319 ± 0.0014	0.4619 ± 0.0033	0.4648 ± 0.0022
iMRW	0.8821 ± 0.0038	0.6892 ± 0.0051	0.6610 ± 0.0046	0.8967 ± 0.0047	0.4715 ± 0.0067	0.4695 ± 0.0054
GraphCDA	0.8361 ± 0.0035	0.5834 ± 0.0057	0.6046 ± 0.0052	0.8555 ± 0.0037	0.4732 ± 0.0056	0.4389 ± 0.0048
DMF	0.8267 ± 0.0043	0.5544 ± 0.0073	0.5394 ± 0.0064	0.8489 ± 0.0059	0.4354 ± 0.0085	0.4429 ± 0.0087
DeepGPA	0.8400 ± 0.0052	0.5503 ± 0.0081	0.5341 ± 0.0088	0.9052 ± 0.0068	0.4643 ± 0.0106	0.4598 ± 0.0108
WDGPA	0.8825 ± 0.0054	0.6797 ± 0.0092	0.6382 ± 0.0089	0.9285 ± 0.0077	0.4830 ± 0.0104	0.4881 ± 0.0095
GDMF	0.9105 ± 0.0067	0.7914 ± 0.0103	0.7612 ± 0.0097	0.9469 ± 0.0085	0.5051 ± 0.0115	0.5013 ± 0.0102

Abbreviations: AUPRC, area under the precision‐recall curve; AUROC, area under the receiver operating characteristic curve; DMF, deep matrix factorization.

### 2.4 GDMF identifies significant de novo GPAs

As shown in Table 4, the GDMF approach has demonstrated superior performance in predicting novel gene‐associated phenotypes, achieving the best results on both maize and human datasets. This is primarily attributed to the integration of GATs and weighted DMF within the GDMF framework, which effectively consolidates multi‐omics data and captures the intricate nonlinear relationships between genes and phenotypes. In contrast, the performance of WDGPA and DeepGPA is second only to GDMF on the human dataset. This may be due to the inclusion of gene expression data from various tissues and experiments in the human dataset, which allows for the reconstruction of attribute data through DMF, yielding a low‐rank representation that better handles gene expression data. However, the maize dataset is relatively smaller and contains more noise and data quality issues, leading to inferior performance compared to the human dataset. The BiRW method exhibits lower performance due to its reliance on gene similarity networks, which are prone to noise. The iMRW method performs well on both datasets, as it can capture key node information through personalized random walks, compensating for partially obscured information by leveraging random walks across multiple subnetworks. GraphCDA and DMF perform better on the human dataset but worse on the maize dataset. This is because the maize dataset features sparser graph structures and sparsest phenotype feature matrices, making it challenging for GraphCDA to learn effective neighborhood information for nodes. DMF struggles with the sparser GPA matrices in the maize dataset, which lack the supplementary information available in other omics data, thus hindering its ability to learn effective low‐dimensional representations from sparse data. Furthermore, the superior results of iMRW, DeepGPA, and WDGPA over BiRW, DMF, and GraphCDA further indicate the advantage of integrating multi‐omics data for the GPA prediction task.

**TABLE 4:** The mean ± standard deviation of results obtained by various methods in predicting de novo GPAs for novel genes on the maize and human datasets.

Methods	Maize			Human		
	AUROC	AUPRC	$F_{\max}$	AUROC	AUPRC	$F_{\max}$
BiRW	0.8124 ± 0.0012	0.5531 ± 0.0033	0.5448 ± 0.0021	0.8917 ± 0.0009	0.3692 ± 0.0023	0.3716 ± 0.0025
iMRW	0.8796 ± 0.0065	0.6813 ± 0.0082	0.6572 ± 0.0074	0.8923 ± 0.0058	0.4314 ± 0.0037	0.4219 ± 0.0043
GraphCDA	0.8392 ± 0.0051	0.5876 ± 0.0069	0.6013 ± 0.0062	0.8821 ± 0.0046	0.3992 ± 0.0029	0.3564 ± 0.0031
DMF	0.8291 ± 0.0078	0.5593 ± 0.0094	0.5428 ± 0.0083	0.8986 ± 0.0075	0.4175 ± 0.0091	0.4232 ± 0.0098
DeepGPA	0.8435 ± 0.0063	0.5662 ± 0.0087	0.5541 ± 0.0079	0.9013 ± 0.0052	0.4317 ± 0.0106	0.4382 ± 0.0074
WDGPA	0.8593 ± 0.0075	0.6171 ± 0.0099	0.6371 ± 0.0098	0.9065 ± 0.0084	0.4380 ± 0.0127	0.4560 ± 0.0115
GDMF	0.9199 ± 0.0085	0.7792 ± 0.0104	0.7631 ± 0.0093	0.9151 ± 0.0071	0.4574 ± 0.0103	0.4607 ± 0.0096

Abbreviations: AUPRC, area under the precision–recall curve; AUROC, area under the receiver operating characteristic curve; DMF, deep matrix factorization; GPAs, genome‐phenome association.

### 2.5 GDMF reveals potential genes associated with important traits

To validate GDMF’s efficacy in identifying potential genes associated with important phenotypes, we excluded known associations from the gene‐phenotype correlation matrix generated by GDMF. Subsequently, we selected the top 11 genes that may be related to photosynthesis and yield traits, as shown in Table 5. These genes were then selected from the gene list in the dataset. These predicted results could be directly or indirectly verified through GO annotations.

**TABLE 5:** Top 11 genes associated with “TO:0000316” (photosynthetic ability, denoted as P for brevity) and “TO:0000371” (yield traits, denoted as Y for brevity) identified by GDMF.

No.	Gene	TO	Evidence	Evidence type
1	GRMZM2G090487	P	GO:0009579	Direct
		Y	GO:0009570	Indirect
2	GRMZM2G135111	P	GO:0009570	Direct
		Y	GO:0009570	Indirect
3	GRMZM2G090528	P	GO:0009704	Direct
		Y	GO:0005515	Direct
4	GRMZM5G865151	P	GO:0005739	Direct
		Y	GO:0034641	Indirect
5	GRMZM5G840279	P	GO:0046872	Direct
		Y	GO:0032502	Direct
6	GRMZM5G890599	P	GO:0007165	Indirect
		Y	GO:0005992	Direct
7	GRMZM2G044457	P	GO:0007165	Indirect
		Y	GO:0007165	Indirect
8	GRMZM2G117392	P	GO:0004672	Indirect
		Y	GO:0007165	Indirect
9	GRMZM5G817625	P	GO:0007165	Indirect
		Y	GO:0005515	Direct
10	GRMZM2G117344	P	GO:1901564	Indirect
		Y	‐	‐
11	GRMZM2G006981	P	‐	‐
		Y	‐	‐

*Note:* “‐” means the contemporary GO annotations do not support the evidence.

Abbreviations: DO, disease ontology; GO, gene ontology; TO, trait ontology.

The gene “GRMZM2G090487” was annotated with “GO:0009507,” “GO:0005759,” and “GO:0036094.” Here, “GO:0009579” represents chloroplasts, the site of photosynthesis, which contains photosynthetic pigments in plants, responsible for capturing light energy and converting it into chemical energy, making it a key organelle for photosynthesis in plants including maize. “GO:0005759” refers to the mitochondrial matrix, which supports the conversion of light energy into chemical energy through enzymatic reactions during maize photosynthesis and energy metabolism, and it releases energy during cellular respiration to meet the plant’s growth and development needs. “GO:0036094” relates to the binding process of photosynthetic pigments. The gene “GRMZM5G865151” was annotated with “GO:0005739,” which defines mitochondria, playing a role in photo synthesis and energy metabolism. The gene “GRMZM2G090528” was annotated with “GO:0036094” and “GO:0009704.” “GO:0009704” is related to the plant’s response to light—specifically, its ability to sense light and perform photosynthesis. The gene “GRMZM2G135111” was annotated with “GO:0036094,” “GO:0009570,” and “GO:0009532.” “GO:0009570” refers to the chloroplast stroma, directly related to photosynthesis, whereas “GO:0009532” refers to plastid stroma, another site of photosynthesis. The gene “GRMZM5G840279” was annotated with “GO:0046872” and “GO:0046914,” which represent metalion binding and transition metalion binding, respectively. These are cofactors for many enzymes and pigments involved in photosynthesis, and transition metal ions play a crucial role in the electron transport chain during photosynthesis. The genes “GRMZM2G044457,” “GRMZM2G090528,” “GRMZM2G117392,” “GRMZM5G817625,” and “GRMZM5G890599” are all associated with “GO:0007165,” which is related to intercellular signal transduction, including light signals, affecting photosynthesis. The gene “GRMZM2G117392” was annotated with “GO:0004672,” referring to protein kinase activity, which participates in various signaling pathways (including light signaling). Photosynthesis is the primary process through which plants produce organic matter and store energy. Maize yield is directly related to its photosynthesis efficiency, photosynthetic capacity, and the photosynthetic activity of its leaves. Improving photosynthesis efficiency often helps increase maize yield. Therefore, genes like “GRMZM2G090487,” “GRMZM5G817625,” and “GRMZM2G117392” indirectly affect maize yield traits. The genes “GRMZM5G840279” and “GRMZM5G817625” were annotated with “GO:0032502,” which defines develop mental processes that directly influence the growth and morphological formation of maize leaves, influencing photosynthetic capacity and energy storage ability, thus affecting maize’s final yield. Moreover, the gene “GRMZM2G090528” was annotated with “GO:0071704” and “GO:0005515.” “GO:0071704” involves organic substance metabolism, including carbohydrates and amino acids. The gene “GRMZM5G817625” was also annotated with “GO:0005515,” which refers to the biocatalytic functions of cytochrome P450 enzymes (P450 enzymes). P450 enzymes are involved in various metabolic pathways, and *DRESH8* may indirectly affect P450 enzyme activity by regulating siRNA expression. Research has shown that wild type maize containing *DRESH8* improved yield traits (including ear length, ear diameter, grain length, grain width, grain thickness, and 100‐grain weight) by 5%–14% compared with gene edited transgenic maize without *DRESH8* [45]. The gene “GRMZM5G890599” was annotated with “GO:0005992,” which is related to the biosynthesis of trehalose, a sugar metabolite involved in maize sugar metabolism. Further, the gene “GRMZM2G135111” was annotated with “GO:1901564” (organic nitrogen compound metabolic process), “GO:0051171” (regulation of nitrogen compound metabolic process), “GO:0044271” (cellular nitrogen compound biosynthetic process), “GO:0006807” (nitrogen compound metabolic process), and “GO:0034641” (cellular nitrogen compound metabolic process). Nitrogen is among the essential nutrients for plant growth, and nitrogen metabolism is a key factor for plant growth and yield. Efficient nitrogen metabolism promotes plant growth and increases yield. The gene “GRMZM2G117344” is also annotated with “GO:1901564” and “GO:0006807,” and the gene “GRMZM5G865151” is annotated with “GO:0034641.” Nitrogen metabolism is crucial for plant growth and yield, as efficacious nitrogen metabolism can promote plant growth and improve yield. Although the gene “GRMZM2G117344” currently lacks annotations related to photosynthesis, and the gene “GRMZM2G006981” is not annotated with information related to photosynthesis or yield traits, this does not necessarily mean that they do not participate in these activities. This behavior remains to be verified by agronomists.

## 3 CONCLUSIONS

In this paper, we proposed an end‐to‐end prediction framework called GDMF, designed to predict the GPA by combining GAT and weighted DMF. The GDMF method leverages graph attention mechanisms to learn attention weights between nodes, and it processes different types of edges through independent convolutional operations, efficaciously capturing the LN structure and key nodes in multi‐omics data. Subsequently, the method adaptively incorporates the global information from different relationship data through weighted DMF, revealing complex nonlinear structures and global dependencies for GPA prediction. The method not only enhances the ability to capture graph structure information and local context but also prevents the smoothing or neglect of key node features due to data scarcity. To overcome the lack of phenotype information, GDMF also incorporates text semantic similarity to enrich the phenotype attribute data. Experimental results on the maize dataset show that GDMF significantly outperformed existing methods across various evaluation schemes, validating its efficacy and superiority in GPA prediction. Through case studies based on gene GO annotations, we identified some genes related to phenotypes, which are worth further verification by agronomists and are expected to be applied in crop breeding and other fields. Future work will explore adaptive weighted matrix selection when processing convolutional attribute data, aiming to more efficaciously incorporate attribute information.

## 4 MATERIALS AND METHODS

This section provides a detailed introduction to the algorithmic principles of the GDMF method, including four key components: The construction of a heterogeneous information molecular network, the calculation of TO text semantic similarity, multisource information learning based on H‐GAT, and weighted DMF for GPA prediction. These components together form the theoretical basis of GDMF, providing solid support for the integration of multi‐omics data and accurate prediction of GPAs. The specific content of these four parts will be introduced in the sequence below.

### 4.1 Heterogeneous information molecular network

To incorporate multi‐omics data, this paper builds a heterogeneous information molecular network (as shown in Figure 1A) consisting of three types of nodes: genes, miRNAs, and TO terms. These nodes are connected via various types of interactions and include multiple node attributes. To standardize the representation of associations and attribute information, we divide the data into two main categories: relationship data, R and attribute data, X. Relationship data are further subdivided into association relationships and interaction relationships. Association relationships capture the associations between different types of nodes, including GPA data, which show the connection between genes and phenotypes. We represent the association matrix between the ith and jth types of molecules as $A_{ij}\in R^{n_{i}\times n_{j}}$, where i, j∈(1,2,...,m) and *i* ≠ *j*. Interaction relationships refer to associations among the same type of nodes, including the similarity between genes or the hierarchical association between TO terms. We represent interaction data for the ith type of molecule as $R_{ii}\in R^{n_{i}\times {\;n}_{i}}$. Attribute data include the intrinsic properties, biological functions, and expression patterns of each node, including gene expression levels, GO enrichment, and semantic similarity of TO texts. The ith type of molecule’s jth attribute is represented by $X_{ij}\in R^{n_{i}\times d_{ij}}$, where $d_{ij}$ is the attribute dimension. By constructing the aforementioned heterogeneous information molecular network, we integrate multi‐omics molecules and their attributes, providing a robust foundation for exploring the complex associations between genes and phenotypes. Table 6 lists the main symbols used.

**TABLE 6:** Symbol definition.

Symbol	Definition
m	Number of molecule types
$n_{i}$	Number of the *i*th type of molecules
R	Relationship data of all molecules
X	Attribute data of all molecules
$A_{ij}$	Association matrix between the *i*th and *j*th types
$R_{ii}$	Interaction matrix for the *i*th type of molecule
$X_{ij}$	The *j*th attribute of the *i*th type of molecule
$\alpha_{vu}$	Attention weight between node *v* and its neighbor *u*
$\chi_{v}^{ini}$	Initial feature representation of the node *v*
$G_{i}^{\text{ini}}$	Initial feature representation of the *i*th type
$W^{r}$	Weight matrix assigned for relationship data matrices

### 4.2 TO text semantic similarity

Plant phenotypes typically refer to various external features and internal traits that can be directly observed (e.g., plant height and size), measured (e.g., yield and disease resistance), or found through laboratory analysis (e.g., nutrient content). However, owing to the scarcity of such data and the difficulty in applying them to computational methods, this paper defines plant phenotypes as terms in the TO, represented by the identifiers (IDs) of TO terms. TO is a specialized vocabulary that describes plant phenotypic traits [46], encompassing distinguishable characteristics and attributes of plants that are either in development or mature. TO is organized in a DAG structure, where each node corresponds to a TO term, and each edge represents the relationships between TO terms. Furthermore, TO follows the true path rule [47], meaning that a child term further refines the functionality of its parent term. This implies that if a gene is annotated with a specific TO term, it is also annotated with the ancestor terms of that TO term; conversely, if a gene is not annotated with a specific TO term, it will not be annotated with any descendant terms of that term. This approach provides a structured framework for the association analysis between genes and phenotypes, helping to deepen the understanding of how genes influence phenotypic characteristics. Figure 2 shows an example of the TO annotation for the maize gene “GRMZM2G315726”: Because the gene “GRMZM2G315726” is annotated with “TO:0000696”, according to the transitive closure rule, the gene is also annotated with the ancestor terms of “TO:0000696” (i.e., “TO:0000489”, “TO:0000281”, “TO:0000277”, and “TO:0000387”). However, since the gene “GRMZM2G315726” is not annotated with “TO:0006008”, it will not be annotated with its descendant term “TO:0000297”. Table 7 provides the definitions of the various TO terms.

**FIGURE 2:**
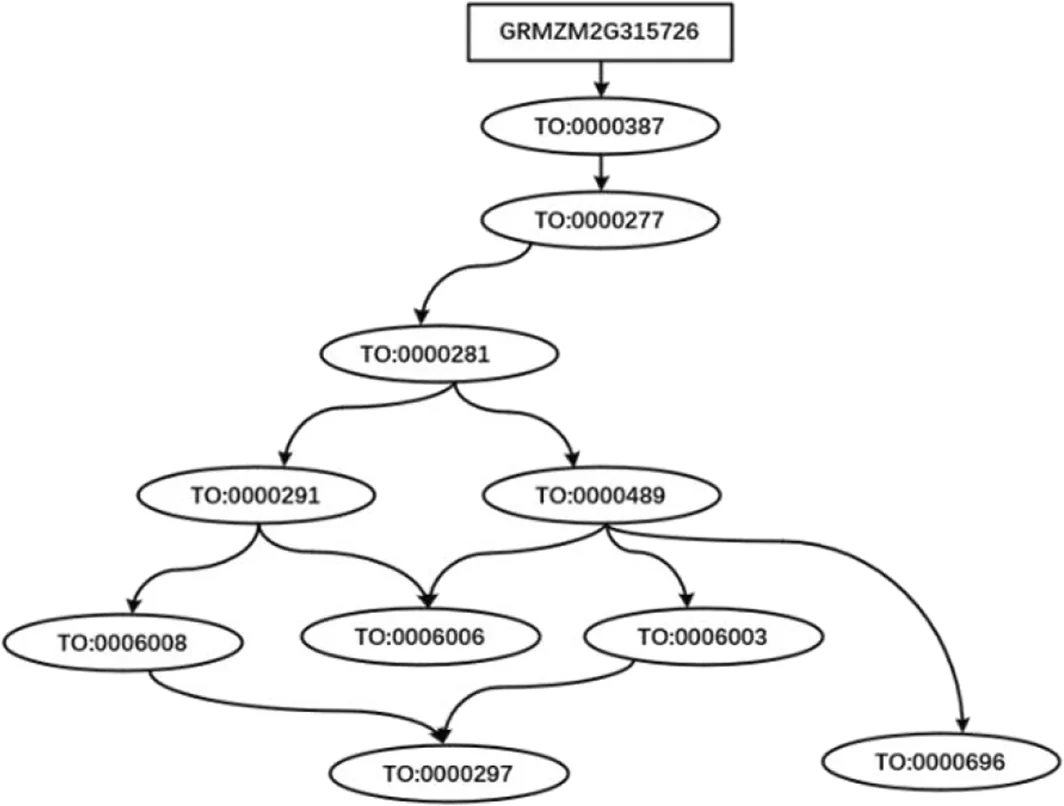
The TO annotation example of the maize gene “GRMZM2G315726”. TO, trait ontology.

**TABLE 7:** Definitions of the various TO terms.

TO term	Meanings
TO:0000387	Plant trait
TO:0000277	Biochemical trait
TO:0000281	Metabolite content trait
TO:0000291	Carbohydrate content
TO:0000489	Carbohydrate composition related trait
TO:0006008	Carbohydrate derivative content
TO:0006006	Monosaccharide content
TO:0006003	Oligosaccharide content
TO:0000297	Glucosamine content
TO:0000696	Starch content

Abbreviation: TO, trait ontology.

TO terms typically include the following attributes: ID, name, description, synonyms, and hierarchical relationships (such as is_a, part_of, and has_part). Previous studies often focus on the hierarchical structure of TO to extract semantic information through these relationships. For example, a deep random walk algorithm might generate a new embedding matrix for each TO node, or transition probabilities might be analyzed to evaluate the semantic similarity between TO terms. However, these methods often overlook the descriptive information contained in the TO terms themselves. TO terms are commonly used in conjunction with the plant and trait ontology (PATO) [48] and the plant ontology (PO) [49]. This indicates that considering only the parent–child relationship structure of TO is insufficient to capture all the information. Whereas TO focuses on the description of traits, particularly the external characteristics and attributes of organisms, PO is concerned with the morphology and developmental processes of plants, especially the definitions of plant organs. PATO combines both PO and TO, offering qualitative and quantitative descriptions of traits, and can be used to integrate the relationships between plant traits and plant structures. Although the hierarchical structure of TO terms is sparse, their textual descriptions contain rich semantic information and relationships with diverse ontology terms. Analyzing these relationships can directly or indirectly reflect associations between TO terms. For example, the description of “TO:0000531” states: “An anther morphology trait (TO:1000022) which is the length (PATO:0000122) of an anther (PO:0009066).” This description defines anther length as a morphology trait of the anther (TO:1000022), with a measurement attribute (PATO:0000122), and also references the anther’s botanical structure (PO:0009066).

Previous studies [33, 44] have often focused on the TO’s hierarchical structure to extract semantic information through these associations. However, these methods often overlook the descriptive information contained in the TO terms. To process the textual descriptions of TO terms, this paper processes the descriptive text of TO terms using the SBERT method. SBERT, through pooling operations, directly generates fixed length sentence embeddings to optimize the computation of sentence–pair similarities. Let $S=\left\{s_{1},s_{2},\text{…},s_{n}\right\}$ be a set of n sentence descriptions, where $s_{i}$ represents the ith TO term’s description. Each sentence is first converted into a sequence of tokens, which may consist of words, subwords, or other linguistic units. Let $T\left(s_{i}\right)=\left\{t_{i1},t_{i2},\text{…},t_{im_{i}}\right\}$ be the token sequence for sentence $s_{i}$, where $m_{i}$ is the number of tokens in sentence $s_{i}$. Each token $t_{ij}$ is converted into a fixed dimensional vector via an embedding layer. Let $E\left(t_{ij}\right)$ be the embedding vector for token $t_{ij}$, which may be pretrained or randomly initialized and learned during training. The embedding representation of sentence $s_{i}$ is as follows:

(1) $$\begin{array}{c} H^{\left(0\right)}\left(s_{i}\right)=\left\{E\left(t_{i1}\right),E\left(t_{i2}\right),\ldots E\left(t_{im_{i}}\right)\right\}, \end{array}$$

where $H^{\left(0\right)}\left(s_{i}\right)$ represents the initial embedding of sentence $s_{i}$. Let $H^{\left(l\right)}\left(s_{i}\right)$ represent the output of the lth layer of the transformer. The sentence representation after the lth transformer layer is as follows:

(2) $$\begin{array}{c} H^{\left(l+1\right)}\left(s_{i}\right)=\text{Transforme}r^{\left(l\right)}\left(H^{\left(l\right)}\left(s_{i}\right)\right) \end{array},$$

where l=0,1,…,L−1, and L is the total number of layers in the transformer encoder. $H^{\left(l\right)}\left(s_{i}\right)$ is the hidden state sequence for sentence $s_{i}$ at the lth layer. Because transformer model [50] specifics were not the focus of this study, detailed formula explanations are omitted. After all encoder layers, SBERT converts the sequence representations into fixed length vectors, usually using pooling operations. Three common pooling methods are CLS pooling, average pooling, and max pooling. This paper uses average pooling as an example:

(3) $$\begin{array}{c} H^{\left(L\right)}\left(s_{i}\right)=\left\{{H_{1}}^{\left(L\right)}\left(s_{i}\right),{H_{2}}^{\left(L\right)}\left(s_{i}\right),...,{H_{m_{i}}}^{\left(L\right)}\left(s_{i}\right)\right\} \end{array},$$

(4) $$E\left(s_{i}\right)=\frac{1}{m_{i}}\sum_{j=1}^{m_{i}}{H_{j}}^{\left(L\right)}\left(s_{i}\right).$$

Finally, a text similarity matrix Sim(S) is built, where each element $\text{Sim}{\left(S\right)}_{ij}$ represents the cosine similarity between sentences $s_{i}$ and $s_{j}$. This generates a new attribute matrix for TO, enhancing TO data:

(5) $$\begin{array}{c} \text{Sim}{\left(S\right)}_{ij}=\frac{E\left(s_{i}\right)\cdot E\left(s_{j}\right)}{\left\|E\left(s_{i}\right)\right\|\cdot \left\|E\left(s_{j}\right)\right\|} \end{array}.$$

### 4.3 Multisource information learning based on H‐GAT

In multisource information learning tasks, node features typically come from multiple heterogeneous data sources, and complex associations exist between the nodes. Although traditional DMF methods can efficaciously extract global features, they may overlook crucial node information, especially LN structures [51], when data are scarce or the sample size is small. This omission can lead to information loss and hamper the extraction of complex features. Moreover, DMF, which primarily extracts global low‐rank patterns, may not fully utilize fine‐grained local topological cues, especially when such structures are sparse or noisy, potentially leading to the smoothing or neglect of important node features [52].

Previous studies have shown that GAT and their variants offer significant benefits in network learning by preserving node level vector embeddings within the network topology. To address the limitations of DMF, we propose a H‐GAT (as shown in Figure 1B) that integrates intra‐type and cross‐type interactions via a type‐specific attention mechanism. The design of H‐GAT follows the biological regulatory cascade rule and propagates information in two stages. First, it aggregates local topological features based on intra type similarities (e.g., gene‐gene similarity). Second, it integrates multisource information through cross‐omics associations (e.g., gene‐TO associations). This hierarchical propagation approach not only preserves the hierarchical structure of biological regulation but also prioritizes high‐confidence biological interactions through dynamic attention weights, thereby suppressing the impact of noisy edges. Specifically, H‐GAT updates the features of a target node by aggregating the features of its neighboring nodes. The attention weight between node *v* and its neighbor *u* is calculated as follows:

(6) $$\begin{array}{c} e_{vu}=\text{LeakyReLU}\left(\alpha^{T}\left[W_{i}\chi_{v}\|W_{j}\chi_{u}\right]\right) \end{array},$$

where $W_{i}$ and $W_{j}$ are the linear transformation matrices for the features of the ith and jth type of nodes, respectively. Specifically, $W_{i}$ is used for transforming the features of node v (which is of type i), and $W_{j}$ is used for transforming the features of node u (which is of type j). $\chi_{v}\in R^{d_{vj}}$ and $\chi_{u}\in R^{d_{uj}}$ are the feature vectors of nodes v and u, respectively. Here, $d_{vj}$ represents the feature dimension of node v before transformation, and $d_{uj}$ represents the feature dimension of node u before transformation. α is the learnable vector for the attention mechanism, and ‖ denotes vector concatenation. The attention weight is then normalized using Softmax as follows:

(7) $$\begin{array}{c} \alpha_{vu}=\frac{\exp\left(e_{vu}\right)}{\sum_{k\in N_{v}}\exp\left(e_{vk}\right)}, \end{array}$$

where $N_{v}$ is the set of neighbors of node v. For each node, its new feature representation is found by the weighted summation of its neighbors’ features and the corresponding attention weights. Specifically, the node feature update formula is as follows:

(8) $$\chi_{v}^{\prime }=\sigma \left(\sum_{u\in N_{v}}\alpha_{vu}W_{j}\chi_{u}\right),$$

where $\alpha_{vu}$ represents the attention weight between node v and its neighbor u, $W_{j}$ is the linear transformation matrix for the node features, and σ is the activation function. In the multi‐head attention mechanism, we use multiple parallel attention heads to calculate the feature updates of nodes, and we concatenate these heads’ outputs to further boost the model’s expressive power:

(9) $$\chi_{v}^{\prime }\left(H\right)={\|\;}_{h=1}^{H}\sigma \left(\sum_{u\in N_{v}}\alpha_{vu}^{h}W_{j}^{h}\chi_{u}\right),$$

where H is the number of attention heads. In the multi‐omics data integration process, different types of nodes interact through various associations. For multisource data, we define separate graph convolution operations for each association, propagating information and updating features through graph convolutions. For instance, gene data first undergo graph convolution based on gene similarity relations, followed by convolution for associations between genes and miRNA, genes and phenotypes. A similar method is used for the miRNA and phenotype data for information propagation and feature updating. The formula is as follows:

(10) $$\begin{array}{c} \chi_{v}^{R_{ii}}=\text{GATConc}\left(\chi_{v}^{\prime H},E_{R_{ii}}\right) \end{array},$$

(11) $$\begin{array}{c} \chi_{v}^{A_{ij}}=\text{GATConc}\left(\chi_{v}^{R_{ii}},E_{A_{ij}}\right) \end{array},$$

where $E_{R_{ii}}$ represents the edge index built based on the $R_{ii}$ relation, $E_{A_{ij}}$ represents the edge index built based on the $A_{ij}$ relation, and GATConc is the graph attention convolution operation. Further, $\chi_{v}^{R_{ii}}$ is the node representation found through convolution based on the interaction, and $\chi_{v}^{A_{ij}}$ is the node representation obtained through further convolution of the associated relationships based on convolutional interactions. This way, the model gradually incorporates information from different data sources, preserving the local structural information of each node, efficaciously avoiding the loss of node features due to information scarcity, and preventing the loss of crucial node information.

After completing multilayer graph convolutions and information aggregation, the node feature representations are mapped to a unified low‐dimensional space and passed through a fully connected layer to find the final node feature representation:

(12) $$\begin{array}{c} \chi_{v}^{\text{ini}}=\text{ReLU}\left(W_{\text{out}}\chi_{v}^{A_{ij}}+b_{\text{out}}\right) \end{array},$$

where $W_{\text{out}}$ and $b_{\text{out}}$ are learnable parameters. For the ith type of molecule, we have $n_{i}$ nodes, with each having an embedding representation. We organize these embeddings into a matrix $G_{i}^{\text{ini}}\in R^{n_{i}\times d}$ as the initial feature representation for the ith type of molecule, where each row represents the embedding vector for one node of this molecule type, and d is the embedding vector’s dimension.

### 4.4 Weighted DMF for GPA prediction

In recent years, DMF has been widely applied for multipath network data fusion. Unlike traditional methods, which mainly explore linear associations between molecules, DMF incorporates global information to capture more complex nonlinear associations, substantially improving the modeling ability for complex molecular interactions. Recent studies have shown that DMF enhances the synergy of global information by optimizing low‐dimensional representations and incorporating weighted strategies, thus augmenting the model’s robustness [53]. Whereas GATs have advantages in modeling local structures and can efficaciously capture local associations between nodes, their ability to model long‐range dependencies or integrate global statistical patterns can be constrained in sparse or fragmented networks. To address this issue, we introduce a weighted DMF technique (Figure 1C) that incorporates global information from different relationship data using an adaptive strategy. This method captures more complex nonlinear structures and global dependencies, compensating for GAT’s shortcomings in global information fusion. For the initial feature representation of molecule i, we learn low‐dimensional representations through a series of neural network layers, as follows:

(13) $$\begin{array}{c} G_{i}=f_{\theta_{N}^{i}}\left(\text{⋯}f_{\theta_{2}^{i}}\left(W_{i2}f_{\theta_{1}^{i}}\left(W_{i1}G_{i}^{\text{ini}}+b_{i1}\right)+b_{i2}\right)\right) \end{array},$$

here $W_{i1}$ and $b_{i1}$ are the weight matrix and bias for the first layer of the i‐type molecule in the neural network, respectively, and $W_{i2}$ and $b_{i2}$ are applied in the second layer. Similarly, this method applies to molecule j. $f_{\theta_{l}^{i}}$ denotes the activation function of the lth layer for the i‐type molecule, and θ represents the model parameters. Based on the principles of MF, we use the learned low‐dimensional representations to reconstruct the association matrix $A_{ij}$ to predict the association between the i‐type molecule and the j‐type molecule:

(14) $$\begin{array}{c} \tilde{A_{ij}}=G_{i}G_{j}^{T} \end{array}.$$

Different biological sub‐networks contain different genetic information, which influences the prediction of GPA in distinct ways. To prevent the loss of global and edge information, we introduce an adaptive weighting strategy that efficaciously incorporates global information from different relational matrices by learning the weighting matrix. This method dynamically adjusts the weight for each relational matrix based on its relative importance, ensuring that when fusing multi‐modal data, local information is preserved, and the global structure is enhanced. Specifically, we minimize the following loss function as the objective function for GDMF:

$$\min_{\Theta }L\left(G,W^{r}\right)=\sum_{A_{ij}\in R}W_{ij}^{r}\|A_{ij}-\tilde{A_{ij}}{\|}_{F}^{2}+\alpha \|W^{r}{\|}_{F}^{2},$$

(15) $$s.t.\;W_{ij}^{r}>0,\sum_{i,j=1}^{m}W_{ij}^{r}=1,$$

where $W^{r}\in R^{m\times m}$ contains the weights assigned to |R| relational matrices. The term $\|W^{r}{\|}_{F}^{2}$ is a regularization term used to prevent trivial weight allocations, and α controls the weights’ complexity and selectivity. Moreover, Θ stores the parameters of the weighted DMF network.

The specific initialization, update, and constraint mechanisms for the weight matrix $W_{ij}^{r}$ are detailed as follows. All learnable weight parameters for relational matrices are uniformly initialized as $W_{ij}^{r}=1/|R|$. This equal‐proportion initialization ensures that all data sources have the same relative importance at the beginning of training, providing an unbiased starting point for subsequent adaptive adjustments. The weight updates are accomplished through optimization of the objective function in Equation (15), with the optimization problem solved using the adaptive moment estimation optimizer. In each iteration, the reconstruction error $\|A_{ij}-\tilde{A_{ij}}{\|}_{F}^{2}$ of relational matrix $A_{ij}$ directly influences the weight update direction, with matrices exhibiting lower reconstruction errors tending to receive higher weights. Simultaneously, the regularization term $\alpha \|W^{r}{\|}_{F}^{2}$ prevents excessive concentration of weight assignments. To ensure stable and interpretable weight assignments, we implement a dual‐constraint mechanism. First, by applying softmax normalization during the optimization process, all weights are constrained to the (0, 1) interval with a sum of 1. Second, the regularization term $\alpha \|W^{r}{\|}_{F}^{2}$ controls weight complexity through hyperparameter α (set to 1.0), preventing over‐reliance on any single data source. This design enables GDMF to dynamically balance contributions from heterogeneous relational matrices.

By minimizing the above equation, we can establish more precise associations between different data types, arriving at a more comprehensive understanding of the complex associations between genes and phenotypes. We use back propagation with ADAM and Softmax batch optimization to adjust the model parameters, ultimately finding the low‐dimensional representations for all molecules. The entire optimization process is described in Algorithm 1.

The computational complexity of the GDMF framework can be systematically analyzed based on its core modules. In the H‐GAT module, the heterogeneous graph contains m types of nodes, with the number of nodes in each type denoted as $n_{i}$. The node feature dimension is unified as d. H‐GAT processes intra‐type relation edges (e.g., gene‐gene similarity) and inter‐type association edges (e.g., gene‐TO association) sequentially. For any edge type (i,j), let the number of edges be $\left|E_{ij}\right|$. Under a single‐head attention mechanism, the complexity of computing attention coefficients and feature aggregation is $O\left(\left|E_{ij}\right|\cdot d_{\text{in}}\cdot d_{\text{out}}\right)$, where $d_{\text{in}}$ and $d_{\text{out}}$ are the input and output dimensions of that layer, respectively. When H attention heads are used, the complexity scales linearly to $O\left(\left|E_{ij}\right|\cdot d_{\text{in}}\cdot d_{\text{ou}t}\cdot H\right)$. Therefore, the overall time complexity of H‐GAT is $O_{\text{GAT}}=O\left(H\cdot \sum_{R_{ii}\in R}\left|E_{ii}\right|{\cdot d}^{2}+H\cdot \sum_{A_{ij}\in A}\left|E_{ij}\right|{\cdot d}^{2}\right)$. Node attribute data X, which serves as the feature input, is included in the linear transformation cost described above. In the weighted DMF module, the initial representation output by H‐GAT, $G_{i}^{\text{ini}}\in R^{n_{i}\times d}$, is used as input. An N‐layer fully connected network learns the low‐dimensional embedding $G_{i}\in R^{n_{i}\times k}$ for each node type, where k is the final embedding dimension. For the ith node type, the forward propagation complexity of this network is $O\left(n_{i}\sum_{l=1}^{N}d_{l-1}d_{l}\right)$, where $d_{0}=d$ and $d_{l}$ is the number of neurons in the lth layer. For all m node types, the total complexity is $O_{G}=O\left(\sum_{i=1}^{m}n_{i}\sum_{l=1}^{N}d_{l-1}d_{l}\right)$. Subsequently, the learned low‐dimensional representations are used to reconstruct the association matrix $\tilde{{\;A}_{ij}}=G_{i}G_{j}^{T}$, with a complexity of $O_{\tilde{A}}=O\left(\sum_{A_{ij}\in A}n_{i}n_{j}k\right)$. The model adaptively weights different relations using a learnable weight matrix $W^{r}\in R^{m\times m}$, whose parameter update complexity is $O_{W}=O\left(m^{2}\right)$. Thus, the total time complexity for one forward training iteration of GDMF is: $O_{\text{forward}}=O_{\text{GAT}}+O_{G}+O_{\tilde{A}}+O_{W}$. The overall training complexity is $O_{\text{total}}=\text{Iter}\cdot O_{\text{forward}}$, where Iter is the number of iterations. The back‐propagation complexity is on the same order as $O_{\text{forward}}$, mainly involving gradient computations for sparse adjacency matrices and dense parameter matrices.

Compared to baseline methods such as WDGPA, the additional overhead of GDMF primarily comes from the $O_{\text{GAT}}$ term. However, because biological molecular networks are highly sparse ($|E|\;\ll \;n^{2}$) and the number of attention heads H is set to a small value (e.g., H=6) based on parameter analysis, the actual computational cost is manageable. The experimental results (as shown in Section 2.3) demonstrate that GDMF significantly outperforms baseline methods on both the maize and human datasets, validating that the computational cost introduced to capture local structure and nonlinear relationships is reasonable and necessary. Furthermore, the model can be adapted to graphs of different scales by adjusting the number of attention heads or employing neighbor‐sampling techniques, indicating good scalability.

ALGORITHM 1 **GDMF algorithm.**

1
**Require:** Heterogeneous molecular network; edge index; training iterations Iter; embedding dimension d; regularization coefficient α; number of multi‐head attention heads H; TO description text S.
**Ensure:** Attention weights $\alpha_{vu}$; N‐layer NN parameters $\Theta ={\left\{W_{il},b_{il}\right\}}_{i=1,l=1}^{m,N}$; Node‐type representations $G=\left\{G_{1},\text{…},G_{m}\right\}$; relationship weight matrix $W^{r}$; association matrix $\tilde{A_{ij}}.$
Build TO text semantic similarity Sim(S) by Equations (1), (2), (3), (4), (5).
Build relationship matrices R.
Build attribute matrices X and incorporate Sim(S).
Randomly initialize Θ.
Initialize $W_{ij}^{r}$ with 1/|R|.
Compute initial embeddings $G_{i}^{ini}$ by Equations (6), (7), (8), (9), (10), (11), (12).
**for** iter = 1 **to** Iter **do**
 Update $G_{i}$ via Equation (13).
 Update $W^{r}$ by softmax.
 Optimize Θ in Equation (15) with ADAM.
**End for Return** $\tilde{A_{ij}}$

### 4.5 Parameter analysis

To analyze the influence of key parameters in the GDMF model, we conducted a parameter analysis experiment on the corn dataset and discussed the results in detail. The number of units in the hidden layers of DMF is a critical parameter affecting the algorithm’s performance. We designed a two‐layer neural network for DMF, where the number of units in the first and second layers are denoted as $d_{1}$ and $d_{2}$, respectively. To study the effect of different combinations of $d_{1}$ and $d_{2}$, we tested $d_{1}$ in the range [64,96] and $d_{2}$ within the set [8,16,24,32,40,48,56,64], respectively.

As shown in Figure 3, due to the imbalance caused by the large number of genes and the small number of TO terms, AUPRC and $F_{\max}$ are less stable compared to AUROC. This is because the precision‐recall curve is more sensitive than the ROC curve. When $d_{1}=96$, the model performs best at $d_{2}=40$, with high values for AUROC, AUPRC, and $F_{\max}$. Further increasing the dimension of $d_{2}$ results in a slight decrease in results. This suggests that excessively high values of $d_{2}$ may introduce redundant information, affecting the stability of the model. When $d_{1}=96$, the model performance is relatively stable, especially at $d_{2}=32$ and $d_{2}=40$, where AUROC and AUPRC are both satisfactory. However, the overall performance fluctuation is larger for $d_{1}=64$, which is likely due to insufficient model complexity, leading to shallow learning of more complex features. Larger values of $d_{1}$ enhance the model’s capacity to capture complex patterns and nonlinear relationships in the data, providing more parameters for finer feature extraction, thus improving the overall performance. Conversely, smaller values of $d_{1}$ may lead to underfitting, where increasing $d_{2}$ can improve performance, but the model’s limited expressive power makes it difficult to stabilize the improvements. Additionally, when $d_{2}$ is too small, the embedding space is too compact to capture complex relationships, resulting in poor generalization and lower accuracy and recall, whereas $F_{\max}$ fails to reach high levels. As $d_{2}$ increases, the embedding space becomes more expansive, allowing the model to learn more features. When $d_{2}\;\ll \;40$, performance improves as $d_{2}$ increases, as the embedding information becomes sufficiently captured when $d_{2}=40$. Overall, we chose $d_{1}=96$ and $d_{2}=40$ for our experiments.

**FIGURE 3:**
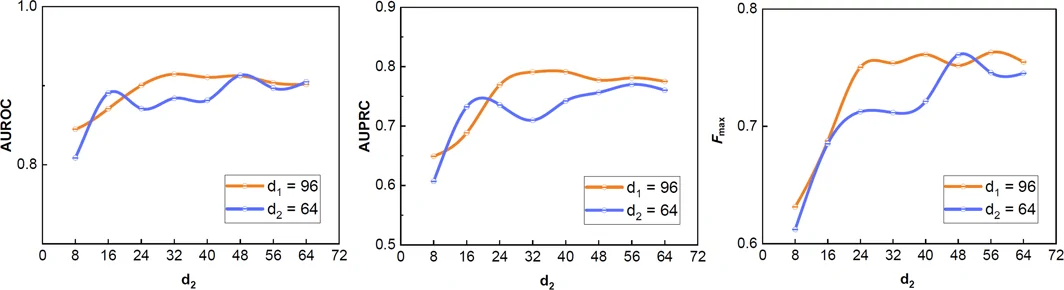
Result of the number of units in the hidden. *d*_1_ and *d*_2_ are the number of the units in the first layer and in the second layer, respectively.

The multi‐head attention mechanism extracts information from multiple subspaces in parallel, enabling the model to capture more complex and diverse relationships. Increasing the number of attention heads can enhance the model’s expressiveness and fitting ability, allowing it to learn from a richer set of features. However, too many attention heads can lead to overfitting and destabilize the model. Therefore, we systematically analyzed the number of attention heads to identify the optimal configuration. As shown in Figure 4, the experimental results indicate that when H=6, the model achieves the best performance in AUROC, AUPRC, and $F_{\max}$. Performance fluctuates to varying degrees as the number of attention heads increases or decreases, with a noticeable drop in performance. Specifically, when H=6, AUROC, AUPRC, and $F_{\max}$ show the best overall performance. This suggests that six attention heads are sufficient to capture the complex feature relationships in the data, enhancing the model’s discriminative ability and generalization capability. At this point, the model can attend to key features in multiple dimensions, helping it adapt better to diverse data patterns. When H≪6, the model’s performance significantly declines. This is because too few attention heads fail to adequately capture the diversity of the data, preventing the model from effectively learning from it. When H≫6, although performance remains stable, the performance starts to fluctuate noticeably at H=12. This is due to the model learning too much redundant information with too many attention heads. Additionally, excessive attention heads can lead to overfitting during training, hindering the model’s ability to generalize effectively. In conclusion, through parameter analysis, we find that $d_{1}=96$ and $d_{2}=40$ offer the best configuration for DMF, as they provide an optimal balance between model complexity and performance. Similarly, the multi‐head attention mechanism achieves optimal results with H=6, which allows the model to effectively capture complex feature relationships without introducing redundancy. These findings highlight the importance of selecting appropriate parameter values for optimizing GDMF and ensuring both model performance and stability.

**FIGURE 4:**
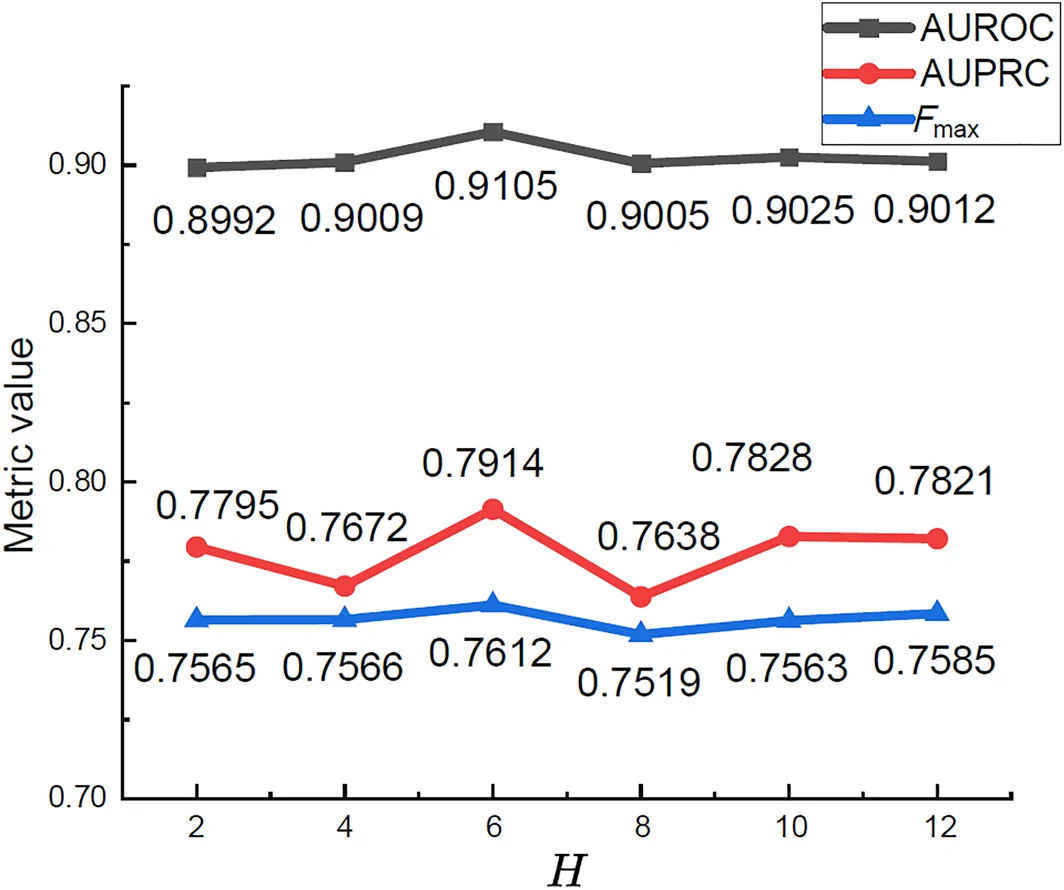
Impact of attention head count on GDMF model performance.

### 4.6 Ablation study

In this study, we designed five variant experiments to assess the contribution of different components of the model. Specifically, GDMF (nS) ignores the TO text semantic similarity and relies solely on the hierarchical information of TO; GDMF (nH) disregards the role of H‐GAT in data integration and directly uses predefined attribute data for DMF to predict GPA; GDMF (nD) excludes DMF and relies only on linear relationships; GDMF (nA) ignores multi‐attribute data matrices and focuses only on the relationships between molecules; GDMF (nM) omits other omics data and considers only GPAs for prediction. The experimental results for GDMF and its variants are shown in Figure 5.

**FIGURE 5:**
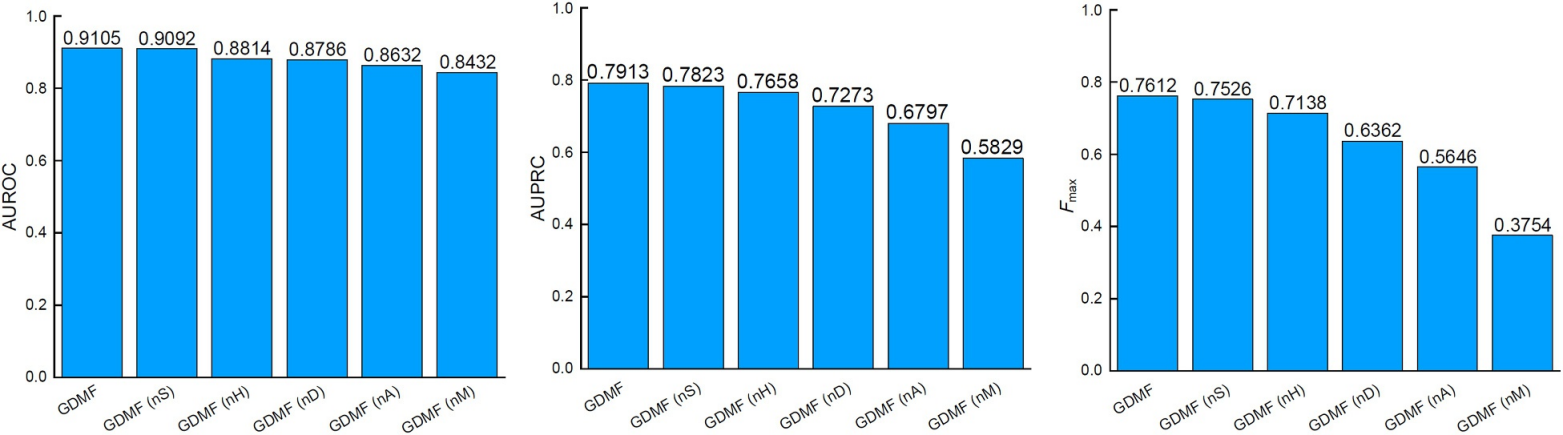
GDMF versus its variants. GDMF (nS) neglects the TO text semantic similarity; GDMF (nH) neglects the role of H‐GAT in data integration; GDMF (nD) excludes DMF and relies only on linear relationships; GDMF (nA) neglects multi‐attribute data matrices; GDMF (nM) omits other omics data and considers only GPAs for prediction. H‐GAT, hierarchical graph attention network; TO, trait ontology.

The experimental results indicate that GDMF outperforms the above variants in terms of AUROC, AUPRC, and $F_{\max}$. Compared to GDMF (nS), the slight performance improvement of GDMF suggests that enriching the TO attribute data is beneficial for enhancing model performance. This finding highlights the importance of semantic similarity in capturing subtle relationships between phenotypes, which can provide additional context for GPA prediction. Given that our GDMF method not only possesses the capability to process graph‐structured data, enabling it to learn rich feature representations from various data sources such as gene and miRNA similarities, but also employs an attention mechanism to dynamically weigh the importance of different data sources. Consequently, the performance improvement brought about by incorporating TO text semantic similarity in GDMF is relatively modest. Although TO text semantic similarity is not the most critical component of GDMF, it serves as a significant supplement for other methods that rely more heavily on a single data source or depend solely on the hierarchical structure of phenotypes. To validate the effectiveness of TO text semantic similarity, we conducted a supplementary experiment. We selected two methods that integrate multi‐omics attribute data—DeepGPA and WDGPA—and introduced our method GDMF. Through five‐fold cross‐validation experiments, we compared the performance of these methods with and without the inclusion of TO text semantic similarity data to assess its impact. As shown in Table 8, the results indicate that the inclusion of TO text semantic similarity data significantly enhances model performance. TO text semantic similarity provides additional information that aids the model in better comprehending the complex associations between TOs, thereby improving predictive accuracy. Specifically, TO text semantic similarity reveals hidden associations between TOs that may not be observable in analyses relying solely on parent‐child hierarchical structure data. The findings demonstrate that TO text semantic similarity offers the model with more contextual information, particularly in multi‐modal data fusion and complex association modeling, significantly boosting model performance.

**TABLE 8:** Performance comparison of different methods with and without TO text semantic similarity data.

Methods	Text_sim	AUROC	AUPRC	$F_{\max}$
DeepGPA	✓	0.8743	0.6684	0.6303
	×	0.8400	0.5503	0.5341
WDGPA	✓	0.9013	0.7175	0.7042
	×	0.8825	0.6797	0.6382
GDMF	✓	0.9105	0.7914	0.7612
	×	0.9092	0.7823	0.7526

Abbreviations: AUPRC, area under the precision‐recall curve; AUROC, area under the receiver operating characteristic curve; TO, trait ontology.

The superior performance of GDMF over GDMF (nH) further confirms the advantage of the multisource H‐GAT in capturing network topology and LN information, which is crucial for efficiently integrating attribute and relational data. The improvement of GDMF over GDMF (nD) highlights the importance of DMF in uncovering nonlinear relationships and integrating global information, which significantly impacts GPA prediction. The higher performance of GDMF compared to GDMF (nA) and GDMF (nM) further demonstrates the positive role of multi‐omics and attribute data in GPA prediction. These results confirm the necessity of using GNN to construct heterogeneous information networks and applying multisource heterogeneous graph attention to learn node neighborhood and graph structure information for multi‐omics data integration. The GDMF model not only integrates global information but also effectively enhances GPA prediction performance by using the attention mechanism to fuse attribute and relational data.

To elucidate the respective limitations of GAT and DMF in GPA prediction and substantiate the necessity of the GDMF framework, systematic perturbation ablation experiments were designed to examine the differential sensitivities of these models to various noise conditions through simulated local structural disruption and global pattern perturbation. Experiments were conducted based on the maize gene‐TO association matrix $A_{14}\in R^{12098\times 155}$ (sparsity 6.42%). Two distinct noise injection strategies were devised: The first category, local structure disruption, involved randomly removing specific proportions (10%–40%) of non‐zero elements from the association matrix, specifically sampling α·nnz(A) positions from the non‐zero index set for zero‐masking, where α∈{0.1,0.2,0.3,0.4}. This operation discretely disrupts the graph connectivity topology, directly impeding local information flow in neighborhood aggregation‐based GNN models. The second category, global pattern disruption, involved injecting Gaussian noise into the matrix as $A^{\prime }=A+\epsilon$ (where $\epsilon ∼N\left(0,\sigma^{2}\right)$, σ∈{0.1,0.2,0.3,0.4}) followed by value truncation, with additional random zero‐masking applied under high‐noise conditions (σ>0.2). This strategy continuously perturbs the matrix value distribution, primarily affecting matrix factorization models that rely on global statistical consistency. These two noise categories respectively target the LN dependency and global statistical consistency of the models, forming dual testing conditions.

In three parallel experimental configurations, the DMF‐only model isolatedly tested MF capabilities, utilizing solely $A_{14}$ as input and optimizing the reconstruction loss $L=\|R-GG^{T}{\|}_{F}^{2},$ through two‐layer nonlinear projections $f_{\theta }:R^{128}\to R^{64}\to R^{40}$, with PCA initialization employed to ensure consistent factorization starting points. The GAT‐only model transformed the association matrix into a bipartite graph comprising 12,253 nodes, constructing bidirectional edge sets. Node features were obtained by separately applying PCA dimensionality reduction to 50 dimensions for the rows and columns of $A_{14}$, ensuring that the GAT received information quantitatively equivalent to that of DMF but presented in GS form, enabling neighborhood aggregation via the attention mechanism $e_{\text{vu}}=\text{LeakyReLU}\left(\alpha^{T}\left[W_{i}\chi_{v}\|W_{j}\chi_{u}\right]\right)$. Notably, local disruption for GAT directly modified the edge_index tensor through edge deletion, whereas global disruption involved adding Gaussian noise to node features to test robustness against feature distribution shifts. The complete GDMF model constructed a heterogeneous graph incorporating multisource nodes and multi‐type edges. AUROC was employed as the primary evaluation metric (supplemented by AUPRC), with performance degradation quantified as $\text{Degradation}=\frac{{\text{AUROC}}_{\text{baseline}}-{\text{AUROC}}_{\text{noise}}}{{\text{AUROC}}_{\text{baseline}}}\times 100\%$ to measure noise sensitivity. Baseline controls were established for each experimental group, with relative degradation rates rather than absolute performance values serving as the focus to eliminate confounding effects arising from inherent performance differences among architectures on clean data.

As shown in Table 9, the DMF‐only model exhibited extreme vulnerability to global pattern disruption; even at 10% noise levels, its AUROC performance declined precipitously from a baseline of 0.8759 to 0.2683, representing a degradation rate of −69.37%. This fragility stems primarily from the extreme sensitivity of its optimization objective to the input matrix value distribution, wherein Gaussian noise disrupts the low‐rank structural assumption underlying the matrix. In contrast, DMF demonstrated relative robustness to local structure disruption, with AUROC declining only to 0.8258 under 40% noise (degradation rate −5.72%), indicating that MF can “complete” local missing information from global distribution patterns.

**TABLE 9:** Performance of DMF‐only baseline under local structure and global pattern disruption.

Noise type	Noise level	AUROC	AUPRC	Degradation (%)
Baseline	0	0.8759 ± 0.0032	0.7906 ± 0.0041	
Local structure disruption	10%	0.8688 ± 0.0028	0.7632 ± 0.0039	−0.81
	20%	0.8658 ± 0.0031	0.7029 ± 0.0052	−1.15
	30%	0.8437 ± 0.0045	0.5669 ± 0.0068	−3.68
	40%	0.8258 ± 0.0056	0.5196 ± 0.0073	−5.72
Global pattern disruption	10%	0.2683 ± 0.0421	0.2237 ± 0.0385	−69.37
	20%	0.3105 ± 0.0389	0.2584 ± 0.0352	−64.55
	30%	0.3539 ± 0.0354	0.2545 ± 0.0321	−59.60
	40%	0.4057 ± 0.0312	0.2727 ± 0.0289	−53.68

Abbreviations: AUPRC, area under the precision‐recall curve; AUROC, area under the receiver operating characteristic curve; DMF, deep matrix factorization.

In stark contrast, as shown in Table 10, the GAT‐only model—without external multisource attributes and lacking multi‐hop neighborhoods due to its bipartite graph structure—proved incapable of exploiting deep GNN aggregation advantages when confronted with local edge removal. At 40% edge removal, AUROC plummeted from 0.7847 to 0.2557 (degradation rate −67.41%), highlighting the high sensitivity and instability of GAT’s neighborhood aggregation mechanism to local connectivity sparsity. Conversely, under global feature noise conditions, GAT exhibited relatively stable performance despite generally lower absolute values, with a degradation rate of merely −37.98% at 40% noise level, indicating that the graph attention mechanism possesses tolerance to feature‐level global perturbations.

**TABLE 10:** Performance of GAT‐only baseline under local structure and global pattern disruption.

Noise type	Noise level	AUROC	AUPRC	Degradation (%)
Baseline	0	0.7847 ± 0.0156	0.4508 ± 0.0123	
Local structure disruption	10%	0.7277 ± 0.0189	0.3796 ± 0.0145	−7.26
	20%	0.3991 ± 0.1245	0.3481 ± 0.0892	−49.14
	30%	0.3026 ± 0.1567	0.2988 ± 0.0956	−61.44
	40%	0.2557 ± 0.0894	0.1402 ± 0.0678	−67.41
Global pattern disruption	10%	0.4645 ± 0.0312	0.0555 ± 0.0089	−40.80
	20%	0.4446 ± 0.0289	0.0559 ± 0.0091	−43.34
	30%	0.4870 ± 0.0267	0.0680 ± 0.0102	−37.94
	40%	0.4867 ± 0.0298	0.0834 ± 0.0115	−37.98

Abbreviations: AUPRC, area under the precision‐recall curve; AUROC, area under the receiver operating characteristic curve; GAT, graph attention network.

As shown in Table 11, the integrated GDMF model successfully amalgamated the strengths of both components, demonstrating balanced robustness across both noise categories: Under 40% local structure disruption, GDMF maintained an AUROC of 0.7764 (degradation rate −14.73%), substantially outperforming GAT‐only’s −67.41%; under 40% global pattern disruption, GDMF degraded by merely −4.87% compared to DMF‐only’s −53.68%, exhibiting stability far surpassing the singular models. Notably, GDMF demonstrated a 0.31% performance improvement under 10% global noise, consistent with low‐dose noise regularization theory. These results indicate that the H‐GAT compensates for DMF’s blind spot regarding local topology, whereas the weighted DMF stabilizes GAT’s predictions under sparse conditions through global low‐rank constraints. This synergistic mechanism enables GDMF to manifest robustness unattainable by individual models in biologically noisy environments.

**TABLE 11:** Comprehensive performance metrics of GDMF framework under local structure and global pattern disruption.

Noise type	Noise level	AUROC	AUPRC	*F*_max_	Degradation (%)
Baseline	0	0.9105 ± 0.0067	0.7914 ± 0.0103	0.7612 ± 0.0097	
Local structure disruption	10%	0.8708 ± 0.0058	0.6414 ± 0.0089	0.6344 ± 0.0072	−4.36
	20%	0.8652 ± 0.0061	0.6979 ± 0.0094	0.6143 ± 0.0081	−4.97
	30%	0.7926 ± 0.0073	0.4449 ± 0.0067	0.4740 ± 0.0063	−12.95
	40%	0.7764 ± 0.0081	0.3813 ± 0.0058	0.4348 ± 0.0054	−14.73
Global pattern disruption	10%	0.9133 ± 0.0062	0.7147 ± 0.0091	0.7603 ± 0.0089	0.31
	20%	0.8719 ± 0.0059	0.6597 ± 0.0086	0.6143 ± 0.0075	−4.24
	30%	0.8685 ± 0.0064	0.6518 ± 0.0082	0.6446 ± 0.0078	−4.61
	40%	0.8662 ± 0.0068	0.5698 ± 0.0074	0.5612 ± 0.0069	−4.87

Abbreviations: AUPRC, area under the precision‐recall curve; AUROC, area under the receiver operating characteristic curve.

## AUTHOR CONTRIBUTIONS

**Ran Duan:** Conceptualization; funding acquisition; methodology; writing—review and editing. **Wei Cao**: Conceptualization; data curation; methodology; software; validation; visualization; writing—original draft; writing—review and editing. **Maozu Guo**: Conceptualization; funding acquisition; investigation; methodology. **Le Tian**: Funding acquisition; methodology; resources. **Lingling Zhao**: Investigation; methodology.

## CONFLICT OF INTEREST STATEMENT

The authors declare no conflicts of interest.

## ETHICS STATEMENT

This article does not contain any studies with human or animal materials performed by any of the authors.

## Data Availability

All source code is available at GitHub webstie (jingjing1728/GDMF).
